# Inducible Volatile Chemical Signalling Drives Antifungal Activity of *Trichoderma hamatum* GD12 During Confrontation With the Pathogen *Sclerotinia sclerotiorum*

**DOI:** 10.1111/1758-2229.70192

**Published:** 2025-09-25

**Authors:** Gareth A. Thomas, József Vuts, David M. Withall, John C. Caulfield, John Sidda, Murray R. Grant, Christopher R. Thornton, Michael A. Birkett

**Affiliations:** ^1^ Protecting Crops and the Environment Rothamsted Research Harpenden UK; ^2^ Biosciences, College of Life and Environmental Sciences University of Exeter Exeter UK; ^3^ School of Life Sciences University of Warwick Coventry UK

**Keywords:** 1‐octen‐3‐one, antagonism, *Sclerotinia sclerotiorum*, *Trichoderma*, volatile organic compounds

## Abstract

The use of beneficial soil fungi or their natural products offers a more sustainable alternative to synthetic fungicides for pathogen management in crops. Volatile organic compounds (VOCs) produced by such fungi act as semiochemicals that inhibit pathogens, with VOC production influenced by physical interactions between competing fungi. This study explores the interaction between the beneficial soil fungus *Trichoderma hamatum* GD12 strain (GD12), previously shown to antagonise crop pathogens such as *Sclerotinia sclerotiorum*, to test the hypothesis that its antagonistic effect is mediated by volatile chemical signalling. In dual‐culture confrontation assays, co‐inoculation of GD12 and *S. sclerotiorum* led to fungistatic interactions after 7 days. VOCs collected from individual and co‐cultures were analysed by gas chromatography–flame ionisation detector (GC‐FID) analysis and coupled GC‐mass spectrometry (GC–MS), revealing significant differences in VOC production between treatments, with VOC production notably upregulated in the GD12 + *S. sclerotiorum* co‐culture. Peak VOC production occurred 17 days post‐inoculation. Synthetic VOC assays revealed several compounds inhibitory to *S. sclerotiorum*, including 1‐octen‐3‐one, which also arrested the growth of other fungal crop pathogens (*Botrytis cinerea*, *Pyrenopeziza brassicae*, and *Gaeumannomyces tritici*). Structural insights into 1‐octen‐3‐one's antifungal activity against *S. sclerotiorum* are also presented. These findings support the hypothesis that the antagonistic properties of *T. hamatum* GD12 against crop fungal pathogens can, in part, be attributed to VOC production. Further research is needed to assess the potential of these semiochemicals as tools for pathogen management in agriculture.

## Graphical abstract

This study found that the beneficial soil fungus *Trichoderma hamatum* GD12 inhibits the fungal pathogen *Sclerotinia sclerotiorum* by producing volatile organic compounds (VOCs). This antifungal activity highlights VOCs including 1‐octen‐3‐one as key contributors. These findings demonstrate fungal VOCs may be promising candidates for managing crop pathogens.

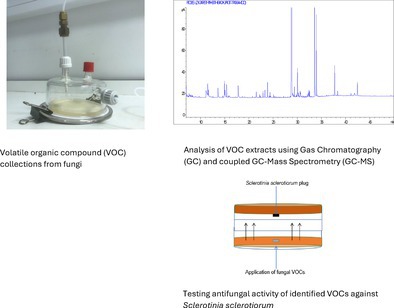

## 1 Introduction

*Sclerotinia sclerotiorum* (Lib.) de Bary (Family: Sclerotiniaceae) is a ubiquitous soil‐borne fungal pathogen, affecting approximately 800 plant species worldwide, including economically important agricultural crops such as carrots, lettuce, sunflower, oilseed rape, and potato (Boland et al. 1994; Bolton et al. 2006). Management of *S. sclerotiorum* on agricultural crops relies mainly on the application of synthetic fungicides (Derbyshire and Denton‐Giles 2016), although the over‐application of fungicides has increased selective pressure, leading to an increase in the frequency of fungicide‐resistant strains (Ma et al. 2009). Alternative methods for controlling *S. sclerotiorum* include the use of crop rotations, which may also be ineffective due to the formation of vegetative sclerotia by *S. sclerotiorum*, which can remain viable in the soil for over 8 years and are resistant to physical, chemical, and biological degradation (Tribe 1957; Adams 1979; Bolton et al. 2006). Moreover, engineering crop resistance toward the pathogen has also proven challenging due to differing pathovars of the pathogen and a lack of resistance in major crops, making breeding programmes a challenge (Bolton et al. 2006; Derbyshire et al. 2022). Therefore, more sustainable approaches for pathogen management on crops that minimise reliance on synthetic fungicides are needed.

Sustainable management strategies for the control of *S. sclerotiorum* include the exploitation of microbial biocontrol agents of the pathogen, including beneficial soil fungi. These decrease the negative potential of pathogens on crops through direct antagonism of the pathogen, competition for resources (e.g., nutrients), or through modification of plant defence responses (Ghorbanpour et al. 2018). *Trichoderma* (Hypocreaceae) is a well‐studied genus of beneficial soil fungi due to its ability to inhibit fungal pathogen development, induce plant defence responses against pathogens, and promote plant growth (Druzhinina et al. 2011; Woo et al. 2023). GD12, a strain of *T. hamatum*, is effective at suppressing the growth of *S. sclerotiorum* in peat‐based microcosms (Ryder et al. 2012; Studholme et al. 2013; Shaw et al. 2016), with the suppressing capability of GD12 requiring the chitinase gene *N*‐acetyl‐β‐glucosaminidase (Ryder et al. 2012). Genome sequencing of *Trichoderma hamatum* (Feng et al. 2025) and specifically GD12 (Studholme et al. 2013) reveals the presence of silent gene clusters, including 32 gene clusters in GD12 comprising Non‐ribosomal peptide synthetases, type 1 polyketide synthases, and terpene synthases, which could be activated in the presence of antagonistic microorganisms in soil, leading to the production of secondary metabolites that are not produced under standard laboratory conditions (Shaw et al. 2016). This antagonism can stimulate the induction of secondary metabolite biosynthetic gene clusters, as evidenced by the induction of genes encoding predicted polyketide synthases (PKSs) and Non‐Ribosomal Peptide Synthetases (NRPSs) clusters during the interaction between *S. sclerotiorum* and *T. hamatum* in peat microcosms (Shaw et al. 2016). However, the causal metabolites involved in these interactions are currently unknown.

Volatile organic compounds (VOCs) are a class of low molecular weight secondary metabolites produced by a wide range of organisms, including soil microorganisms, which contribute to their ability to compete against neighbouring organisms for resources in soil (Garbeva and Weisskopf 2020; Weisskopf et al. 2021). The ability of VOCs to travel between gas‐ and water‐filled pockets in soil classifies them as long‐distance messengers, compared to non‐volatile secondary metabolites, which may be drivers of more local interactions (Kai et al. 2016; Schulz‐Bohm et al. 2017; Westhoff et al. 2017). Microbial VOCs are involved in a range of biological activities, including direct inhibition of pathogenic microorganisms (Fernando et al. 2005), induced plant defence against pathogens (Ryu et al. 2004) and plant growth promotion (Ryu et al. 2003). Several studies indicate *Trichoderma* VOCs can specifically play an inhibitory role against a range of fungal pathogens (Amin et al. 2010; Stoppacher et al. 2010; Jeleń et al. 2014; Meena et al. 2017; Wonglom et al. 2020). These biological activities highlight the potential for microbial VOCs to be used as effective alternatives to pesticides and fertilisers (Thomas et al. 2020, 2023).

Whilst beneficial soil microbes can produce VOCs when grown axenically under standard laboratory conditions, they exist in complex communities within the soil matrix. Genome sequencing of fungal species indicates that many secondary metabolite gene clusters are silent and telomeric under standard laboratory conditions and may require specific cultivation conditions to activate them, including stress inducing or co‐culturing with different species of microorganisms (Scherlach and Hertweck 2021). Experimentally reproducing a more natural microbe interaction environment is feasible through inoculating different species of microorganisms within the same confined space. Such an approach may stimulate the antagonism that activates silent gene clusters, and hence facilitate the discovery of novel, bioactive compounds. It has been observed that ornesillic acid production was uniquely induced through co‐culturing of *Streptomyces* and *Aspergillus* (Schroeckh et al. 2009). This study subsequently led to an expansion in the discovery of novel secondary metabolite production through microbial co‐culture (Knowles et al. 2022). For example, in *T. harzianum*, co‐culture with the endophyte *Talaromyces pinophilus* led to changes in secondary metabolite production relative to monoculture controls (Vinale et al. 2017). The majority of these studies focus on changes in non‐volatile compound production during physical interactions; however, a growing body of evidence suggests co‐culturing can also induce VOC production, with examples from fungal‐fungal (Hynes et al. 2007; Evans et al. 2008; El Ariebi et al. 2016; Guo et al. 2019; O'Leary et al. 2019), bacterial‐bacterial (Tyc et al. 2015, 2017) or fungal‐bacterial (Albarracín Orio et al. 2020) interactions.

Here, we aimed to determine the role of VOCs in the biocontrol capabilities of *T. hamatum* GD12 against *S. sclerotiorum*. We demonstrate: (i) quantitative and qualitative changes in VOC production by *T. hamatum* occur during confrontation with *S. sclerotiorum*; (ii) temporal changes in VOC production during confrontation occur, with maximal induction day 17 post inoculation; (iii) VOCs produced by *T. hamatum* have antifungal activity against *S. sclerotiorum*; (iv) identification of 1‐octen‐3‐one, which completely inhibits the growth of *S. sclerotiorum* as well as other agriculturally important fungal pathogens; and (v) the structural features required for the antifungal activity of 1‐octen‐3‐one against *S. sclerotiorum*. This work highlights the power of using *Trichoderma*‐pathogen co‐culture to reveal cryptic chemistries encoding bioactive VOCs for use as pathogen management tools in agriculture.

## 2 Materials and Methods

### 2.1 Dual‐Culture Confrontation Assays

*Trichoderma hamatum* GD12 isolated from a potato field (Great Down, Devon, UK) (Thornton, pers. comm.) and *S. sclerotiorum* isolate 1 (isolated on oilseed rape petal, ADAS Rosemaund, Herefordshire, UK) (West, pers. comm.), used in the study were maintained on Potato Dextrose Agar (PDA) (15 g Bacteriological Agar No. 2, LabM, UK; 24 g Potato Dextrose Broth (PDB), Sigma, UK; 1000 mL distilled H_2_O) slopes in sterile, screw‐capped plastic vials (ThermoScientific, UK). Circular plugs (5 mm diam.) were cut using a sterilised cork‐borer (Sigma, UK) from the leading edge of mycelia of 3‐day‐old PDA plates of *T. hamatum* GD12 or *S. sclerotiorum* isolate 1. Each experiment comprised (a) a control, containing uninoculated growth media (PDA), (b) *T. hamatum* GD12 co‐cultured against itself (self‐challenged), (c) *S. sclerotiorum* isolate 1 co‐cultured against itself (self‐challenged), and (d) *T. hamatum* GD12 challenged against *S. sclerotiorum* (co‐culture) (*n* = 4). Plugs from individual strains of *T. hamatum* were placed approximately 80 mm away from plugs of *S. sclerotiorum* on fresh PDA plates (90 mm) and grown for 7 days under a 16 h/8 h fluorescent light/dark photoperiod at 24°C until required for dynamic headspace collection experiments (detailed below).

### 2.2 Dynamic Headspace Collection (Air Entrainment)

PDA plates containing 7‐day‐old fungal cultures (see above) were enclosed individually in glass entrainment vessels (12 cm diam. × 6 cm height). Charcoal‐purified air (flowrate 600 mL/min) was pushed into each entrainment vessel and drawn (flowrate 500 mL/min), ensuring a positive pressure (100 mL/min) throughout the system. Air was drawn through a glass tube containing Porapak Q (50 mg, 50/80 mesh, Supelco, Bellefonte, PA) held with two plugs of silanized glass wool, for 20 h at ambient temperature (Pye volatile collection kits, Kings Walden, UK). Before each collection, glass vessels were washed with Teepol detergent, rinsed with distilled water, washed with acetone (ThermoFisher, UK), and then placed in a modified heating oven (180°C) for a minimum of 2 h. Charcoal filters (10–14 mesh, 50 g) (Sigma, UK) were conditioned prior to each experiment by attaching them to a supply of nitrogen in a modified heating oven (150°C) under a constant stream of nitrogen. VOC collections were performed under a 16 h/8 h light/dark photoperiod at 24°C. Porapak Q traps were cleaned by washing with freshly redistilled diethyl ether (2 mL) and heated to 132°C for a minimum of 2 h under a stream of nitrogen. Following collections, VOCs were eluted from the Porapak Q traps with freshly redistilled diethyl ether (750 μL) into 1.1 mL pointed vials (ThermoScientific, Germany), capped with an 8 mm Chromacol screw cap vial lid (ThermoScientific, Germany) with an 8 mm Silicone Red PTFE Septa (Kinesis, UK). The eluent was concentrated to 50 μL under a gentle stream of nitrogen and stored at −20°C prior to further analysis.

### 2.3 Time‐Course VOC Collection Experiment

Solid‐phase microextraction (SPME) was selected as the method for VOC analysis for time‐course experiments rather than dynamic headspace collection, as preliminary experiments showed repeated dynamic headspace collections of VOCs from fungal cultures led to drying out of growth media, impacting fungal growth. An SPME (100 μM Polydimethylsiloxane (PDMS) fibre, Supelco, UK) was introduced into the GC thermal desorption injector port to desorb for 10 min (temperature of injector = 250°C). The SPME fibre was inserted through a clean septum and exposed to the headspace of the fungal culture within a clean glass entrainment vessel (12 cm diam. × 6 cm height) for 1 h. SPME samples were taken at 1, 2, 3, 4, 5, 6, 7, 10, 17, and 24 days post inoculation (dpi) from cultures of (a) self‐challenged *S. sclerotiorum* isolate 1, (b) self‐challenged *T. hamatum* GD12, or (c) GD12 co‐cultured with *S. sclerotiorum*. The first sampling timepoint (day 1 post inoculation) of the experiment was used as a baseline, with 80 mm of distance between the fungal mycelia. At day 2, the distance between the self‐challenged GD12 treatments was 19 to 24 mm, and 7 to 15 mm for the GD12 co‐cultured with *S. sclerotiorum* treatments. Self‐challenged *S. sclerotiorum* treatments had already initiated contact by this stage of sampling. By day 3, contact between mycelia across all treatments had established.

### 2.4 Gas Chromatography—Flame Ionisation Detector (GC‐FID) Analysis

Air entrainment samples were analysed on an Agilent 6890 GC equipped with a cool on‐column injector, an FID and a HP‐1 bonded‐phase fused silica capillary column (50 m × 0.32 mm i. d. × 0.52 μm film thickness). The oven temperature was set at 30°C for 0.1 min, then increased at 5°C/min to 150°C for 0.1 min, then at 10°C/min to 230°C for a further 25 min. The carrier gas was hydrogen. VOCs adsorbed on SPME fibres were thermally desorbed by inserting the fibre directly into the OPTIC Programmable Temperature Vaporisor (PTV) unit (30 ‐ > 250*°*C ballistically at a rate of 16*°*C/s).

### 2.5 Coupled GC‐Mass Spectrometry (GC–MS)

An Agilent Mass Selective Detector (MSD) 5973 coupled to an Agilent 6890N GC (fitted with a non‐polar HP1 column 50 m length × 0.32 mm i. d. × 0.52 μM film thickness, J & W Scientific) was used for analysis. Sample injection was via cool‐on column and MS ionisation was by electron impact at 70 eV at 220°C. The GC oven temperature was maintained at 30°C for 5 min and then programmed at 5°C/min to 250°C, run time 70 min. Tentative identifications were made by comparison of mass spectra with NIST11 mass spectral database and by comparison of GC retention indices (Kováts Index, KI) and confirmed by co‐injections with authentic standards. The KIs of the compounds were calculated relative to the retention times of the homologue series of n‐alkanes (C7–C22), on a HP‐1 column.

### 2.6 Chemicals

1‐Pentanol (99%), 1‐octen‐3‐ol (98%), 1‐octene (98%), 2‐undecanone (99%), 2‐octanone (98%), 3‐octanone (99%), 2‐pentylfuran (≥ 98%), 6‐*n*‐pentyl‐2H‐pyran‐2‐one (6‐PAP) (≥ 96%), 1‐octen‐3‐one (96%) and 2‐heptanone (98%) were all purchased from Sigma‐Aldrich, UK. Diethyl ether (99.5%) was purchased from Fisher and redistilled before use.

### 2.7 Synthetic Compound Assays

Synthetic standards of VOCs identified from air entrainments of *T. hamatum* GD12 were applied to sterile qualitative filter paper (6 mm) (Whatman, UK) and placed onto a Petri dish containing PDA. On a fresh plate of PDA, a plug (5 mm diam.) of *S. sclerotiorum* was inoculated, and the plate was inverted over another plate containing the filter paper with the synthetic VOC sample, and the two plates were then sealed with tape, ensuring no physical contact between the VOC sample and the pathogen (Inverted plate bioassay, Figure S1). Solutions of VOCs were prepared in freshly redistilled diethyl ether to ensure that a 20 μL application of solution gave a dose of 45.5 μM. This dose was decided based on preliminary experiments, where 5 μL of each compound was applied neat to a sterile filter paper, and the dose selected was based on the least inhibitory compound (1‐pentanol; 5 μL of which equates to 45.5 μM). This dose is also similar to that used in previous studies (Tyc et al. 2015). For compounds demonstrating significant (*p* < 0.05) antifungal activity relative to control treatments when applied at 45.5 μM, doses onto filter discs were diluted to 22.75, 11.125, 4.55, 2.275, 0.91 and 0.455 μM in freshly redistilled diethyl ether from a stock solution until no further inhibition was observed. Mycelial measurements from *S. sclerotiorum* were taken using a 30 cm ruler after 72 h. From the diameter of *S. sclerotiorum*, the area of the colony was calculated as *πr* ^2^, where “*r*” is equal to the radius of the colony. Control plates contained a sterile filter paper disc with 20 μL of freshly redistilled diethyl ether alone.

To evaluate the antifungal activity of 1‐octen‐3‐one against *Botrytis cinerea*, *Pyrenopeziza brassicae* and *Gaeumannomyces tritici*, fungal cultures were maintained under the following growth conditions:

*Botrytis cinerea* (isolated from shop‐bought strawberries, UK) was cultured on PDA and incubated in the dark at 21 °C. For inverted plate bioassays, 5 mm diameter plugs were inoculated onto fresh PDA, and bioassays were performed as described above.

*Gaeumannomyces tritici* (isolate 17LH(4)19d1, Rothamsted Farm, UK (Chancellor et al. 2024)) was cultured on PDA and incubated in the dark at 21 °C. For inverted plate bioassays, 5 mm diameter plugs were inoculated onto fresh PDA, and bioassays were performed as described above.

*Pyrenopeziza brassicae* (isolate 112‐16, Northumberland, UK) was cultured on 3% malt extract agar (MEA; 30 g malt extract, 15 g agar) in the dark at 18 °C on 9 cm Petri dishes. After 21 days, conidia were harvested by adding 2 mL of sterile 0.1% Tween 80 and gently scraping the colony surface with a sterile inoculation loop. The resulting suspension was filtered through sterile Miracloth and diluted to 1 × 10⁶ spores mL⁻¹ using sterile 0.1% Tween 80. For inverted plate bioassays, 20 μL of conidial suspension was added onto fresh MEA, and bioassays were performed as described above.

Inverted plate bioassays for all species were performed using a 45.5 μM dose of 1‐octen‐3‐one, ensuring no physical contact between the fungal cultures and the compound.

### 2.8 Statistical Analysis

For comparison of GC analysis from confrontation assays, peak area values were individually measured (Agilent Chemstation) and log_10_‐transformed. An adjustment of 0.001 was applied to account for values recorded as zero. Statistical comparison of compounds present in both mono‐ and co‐culture treatments was analysed using an unpaired Student's *t*‐test assuming equal variances, one variate with grouped factor. To establish the antifungal activity of selected VOCs, mycelial areas were statistically compared across. Inverted plate bioassays for all species were performed using a 45.5 μM dose of 1‐octen‐3‐one, ensuring no physical contact between the fungal cultures and the volatile compound. Treatments using one‐way analysis of variance (ANOVA), followed by Tukey's Honest Significant Difference test at the *p* < 0.05 level, where multiple comparisons were required. Genstat (v21, VSN International, Hemel Hempstead, UK) was used for all statistical analyses.

## 3 Results

**FIGURE 1:**
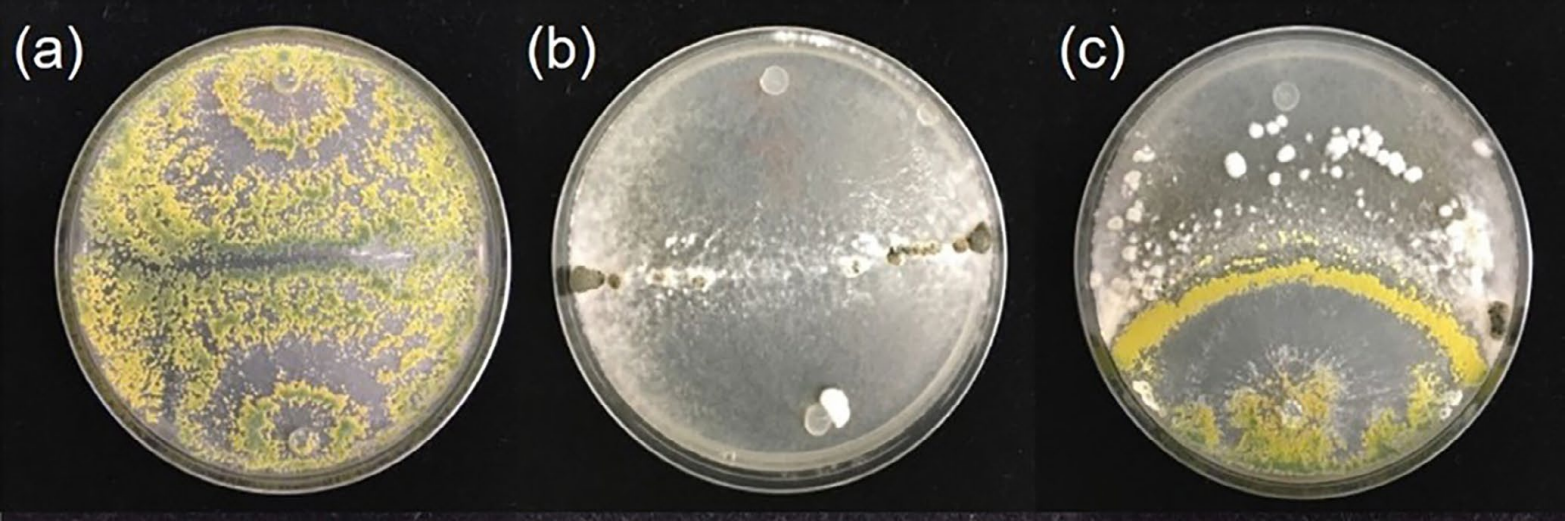
Dual‐culture confrontation assays of (a) self‐challenged *Trichoderma hamatum* GD12 strain; (b) self‐challenged *Sclerotinia sclerotiorum* (c) co‐culture of *S. sclerotiorum* (top) and *T. hamatum* GD12 (bottom).

Dual‐culture confrontation assays demonstrated fungistatic interactions between *T. hamatum* GD12 when confronted with *S. sclerotiorum* after 7 days of growth (Figure 1). This was accompanied by the formation of yellow spores by *T. hamatum* GD12 in the interaction zone between the two fungi.

**FIGURE 2:**
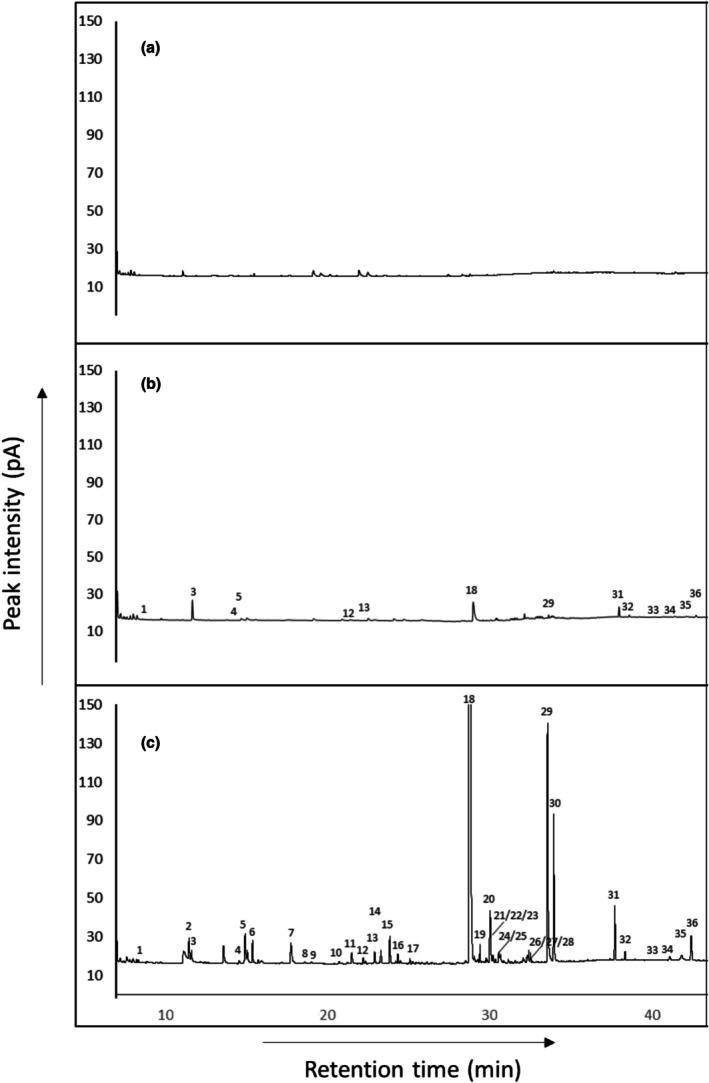
Representative gas chromatographic analysis of volatile organic compounds (VOCs) collected by air entrainment from 7‐day‐old cultures of (a) self‐challenged *Sclerotinia sclerotiorum*, (b) self‐challenged *T. hamatum* GD12, and (c) *T. hamatum* GD12 co‐inoculated with *S. sclerotiorum*. For an explanation of peak numbers, see Table 1.

Significant quantitative and qualitative changes in VOC production compared to self‐challenged GD12 treatments were observed when *T. hamatum* GD12 was co‐cultured with *S. sclerotiorum* (Figure 2; Table 1). Production of 6‐pentyl‐2H‐pyran‐2‐one (6‐PAP) (compound 18, Figure 2) dominated the headspace of GD12 co‐cultured with *S. sclerotiorum*, with mean production of 6‐PAP significantly greater in these co‐cultures compared to self‐challenged controls (*p* < 0.001) (Table 1). Of the 36 compounds detected, eight of which were confirmed by co‐injection, 22 were unique to GD12‐*S. sclerotiorum* co‐cultures, suggesting that the VOCs were either biosynthesised *de novo* in the presence of *S. sclerotiorum* or produced below detectable limits of the GC in GD12 monocultures, including 2‐pentylfuran (Figure 2; Table 1).

**TABLE 1:** Composition of VOCs from dynamic headspace collections of 7‐day‐old cultures of self‐challenged *Trichoderma hamatum* GD12, or GD12 co‐inoculated with *Sclerotinia sclerotiorum* (*n* = 4) (mean peak area ± SE). Data were analysed by Student's *t*‐test (*p* < 0.05). Values in bold indicate compounds that showed statistically significant differences between treatments, or those that were uniquely present in a single treatment. KIs on a non‐polar HP‐1 GC column. The identity of compounds marked with an asterisk were confirmed by GC peak enhancement via co‐injection with authentic standards.

Peak no.	Compound	KI	GD12 vs. *S. sclerotiorum*	GD12 vs. GD12	*p*
1	1‐Pentanol*	**754**	**7.29 (± 1.85)**	2.42 (±0.37)	0.016
2	2(5H)‐Furanone	**871**	**95.43 (± 24.50)**	n.d.	NA
3	2‐Heptanone*	**876**	26.08 (±4.82)	26.47 (±7.6)	0.813
4	1‐Octen‐3‐one*	**959**	8.76 (±3.92)	11.49 (±9.23)	0.606
5	3‐Octanone*	**969**	50.98 (±17.87)	41.25 (±22.83)	0.439
6	2‐Octanone*	**972**	**29.00 (±6.52)**	n.d.	NA
7	2‐Pentylfuran*	**981**	**142.00 (± 48.95)**	n.d.	NA
8	No i.d.	**990**	**4.65 (±0.44)**	n.d.	NA
9	No i.d.	**995**	**6.90 (±1.81)**	n.d.	NA
10	No i.d.	**1049**	**30.67 (±13.89)**	n.d.	NA
11	No i.d.	**1074**	**8.98 (±1.59)**	n.d.	NA
12	No i.d.	**1137**	25.29 (±12.24)	20.34 (±13.59)	0.51
13	2‐n‐Heptylfuran	**1182**	12.60 (±3.31)	7.61 (±4.01)	0.278
14	Cyclodecanone	**1217**	**28.09 (±9.8)**	n.d.	NA
15	2‐Butyl‐cyclodecanone	**1235**	**71.50 (±29.64)**	n.d.	NA
16	No i.d.	**1252**	**53.66 (±34.92)**	n.d.	NA
17	2‐Undecanone*	**1276**	**9.00 (±1.54)**	n.d.	NA
18	6‐Pentyl‐2H‐pyran‐2‐one*	**1429**	**7596.41 (±1617.76)**	58.04 (±20.08)	0.001
19	No i.d.	**1457**	**27.35 (±5.69)**	n.d.	NA
20	No i.d.	**1488**	**90.47 (±24.10)**	n.d.	NA
21	No i.d.	**1497**	**14.21 (±4.25)**	n.d.	NA
22	No i.d.	**1505**	**8.65 (±1.61)**	n.d.	NA
23	No i.d.	**1518**	**8.12 (±2.04)**	n.d.	NA
24	No i.d.	**1565**	**12.03 (±5.38)**	n.d.	NA
25	No i.d.	**1608**	**6.67 (±0.54)**	n.d.	NA
26	No i.d.	**1625**	**11.33 (±2.90)**	n.d.	NA
27	No i.d.	**1632**	**14.44 (±3.66)**	n.d.	NA
28	No i.d.	**1639**	**12.89 (±3.51)**	n.d.	NA
29	No i.d.	**1710**	**334.61 (±103.98)**	5.19 (±3.42)	0.004
30	No i.d.	**1739**	**98.07 (±42.20)**	n.d.	NA
31	No i.d.	**2018**	**68.41 (±14.13)**	7.67 (±2.77)	0.002
32	No i.d.	**2057**	**9.81 (±2.14)**	1.45 (±0.67)	0.003
33	No i.d.	**2198**	2.40 (±0.77)	0.65 (±0.39)	0.105
34	No i.d.	**2208**	**8.00 (±1.56)**	1.60 (±0.69)	0.006
35	No i.d.	**2240**	11.00 (±3.56)	1.55 (±0.78)	0.043
36	No i.d.	**2266**	**43.73 (±8.74)**	2.85 (±0.72)	0.001

Abbreviation: n.d., not detected.

**FIGURE 3:**
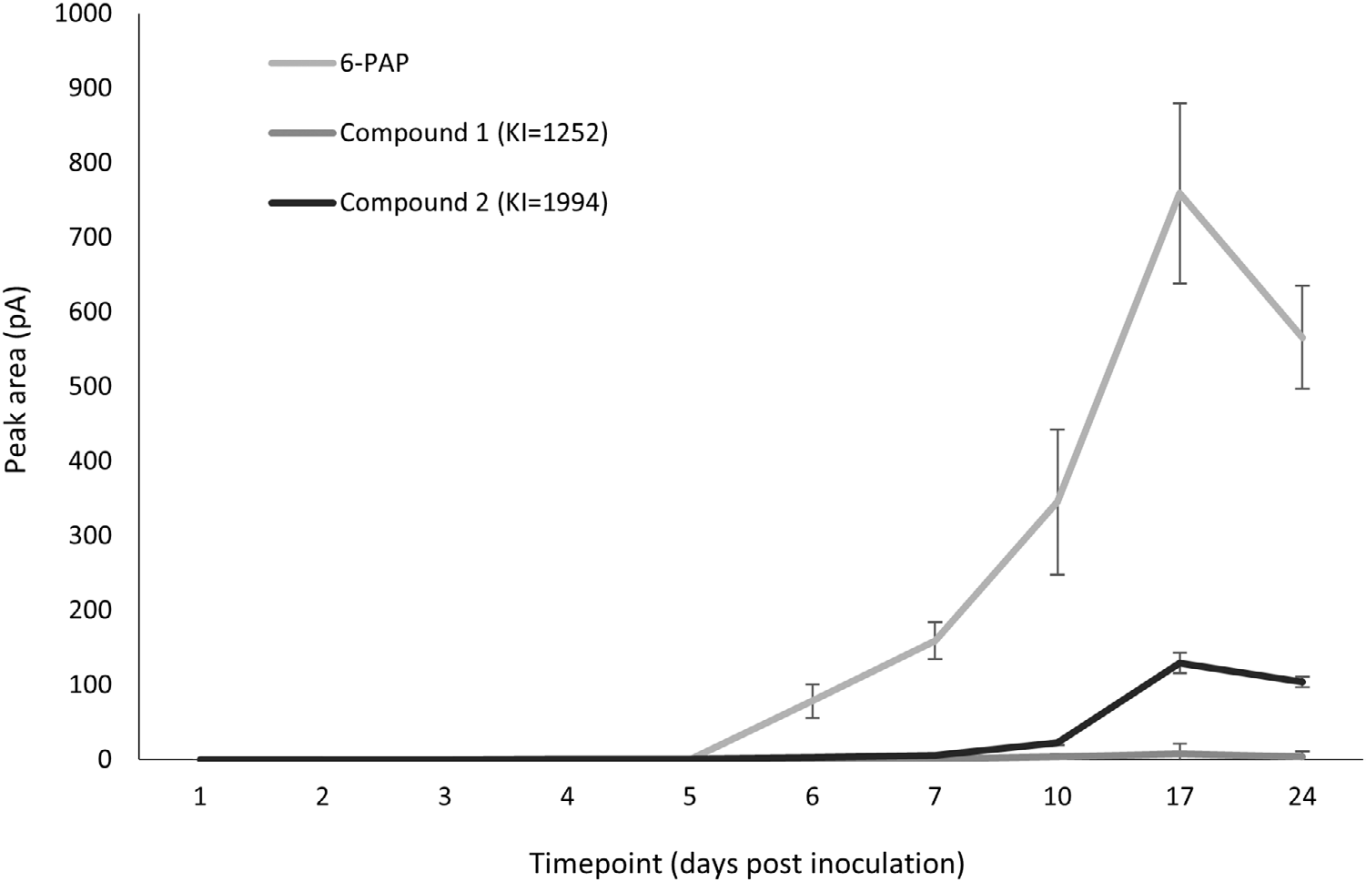
Production of VOCs in treatments of *Trichoderma hamatum* GD12 and *Sclerotinia sclerotiorum* co‐culture treatments over the course of 24 days for three VOCs. Bars represent the peak area value of each VOC (± SD) (*n* = 3).

As co‐culturing *T. hamatum* GD12 with *S. sclerotiorum* led to significant increases in VOC production, the temporal dynamics of *T. hamatum* GD12 VOC production were investigated. For three compounds detected by SPME, peak induction was greatest at day 17 post‐co‐culture, each subsequently decreasing by day 24 (Figure 3). Each compound showed similar trends in production across the different time points. In GD12 co‐cultured with *S. sclerotiorum* treatments, compound 1 (KI = 1252) was not detected in the headspace until day 6. By day 10 there was an increase in production, which further increased at day 17, subsequently decreasing to day 24.6‐PAP shows a similar trend. Compound 2 (KI = 1994), detected by day 10, increased until day 17, and then decreased by day 24.

**FIGURE 4:**
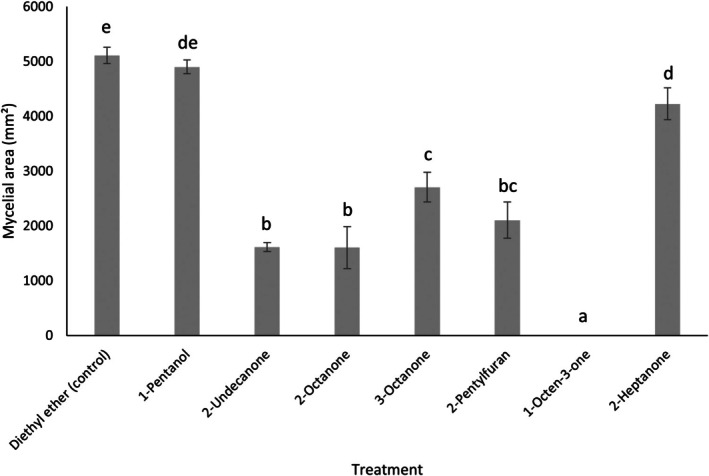
Antifungal activities of selected VOCs on the growth of *Sclerotinia sclerotiorum*. *S. sclerotiorum* was incubated with selected VOCs at 45.5 μM doses and the inhibition rates were calculated relative to control plates (exposed to diethyl ether alone) after 3 days. Bars represent the mean mycelial area of *S. sclerotiorum* upon exposure to each VOC (± SD) (*n* = 3). Different letters indicate significant differences between treatments according to Tukey's multiple comparisons test (*p* < 0.05). Y axis represents mycelial area (mm^2^).

In preliminary experiments with *T. hamatum* VOCs and *S. sclerotiorum*, 6‐PAP had no detectable inhibitory activity against *S. sclerotiorum* (*F*_2,6_ = 2.17, *p* = 0.195) (Figure S2) and was therefore excluded from further bioassays. The mycelial area of *S. sclerotiorum* differed significantly depending on the specific VOC applied at the highest dose (45.5 μM), indicative of differences in their inhibitory activities (*F*_7,16_ = 171.36; *p* < 0.001; *n* = 3) (Figure 4). The mycelial area of *S. sclerotiorum* exposed to 1‐pentanol was not significantly different to solvent control treatments (*p* > 0.05), and 2‐heptanone treatments were not significantly different to 1‐pentanol treatments (*p* > 0.05), so these compounds were not tested at reduced doses. 2‐Octanone and 2‐undecanone demonstrated similar levels of inhibition, while 2‐octanone was significantly more inhibitory than 3‐octanone. Strikingly, 1‐octen‐3‐one demonstrated effectively complete growth inhibition of *S. sclerotiorum*. Importantly, this antifungal activity was effective against other economically important fungal pathogens (*Botrytis cinerea*, *Pyrenopeziza brassicae* and *Gaeumannomyces tritici*) (Figure S3).

**FIGURE 5:**
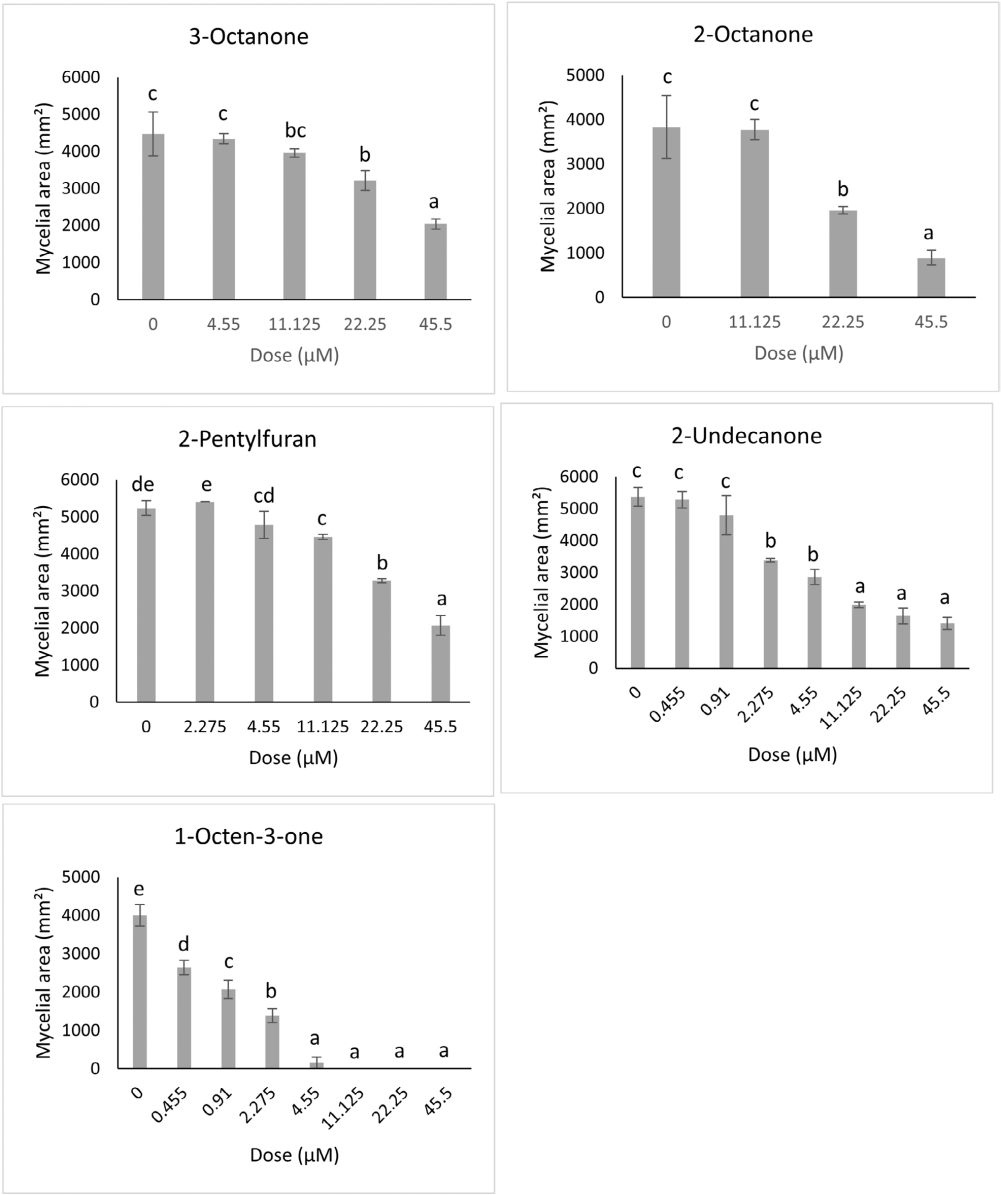
Antifungal activities of selected VOCs on the growth of *Sclerotinia sclerotiorum*, at reduced doses. Bars represent the mean mycelial area of *S. sclerotiorum* upon exposure to each VOC (± SD) (*n* = 3). Different letters indicate significant differences between treatments according to Tukey's multiple comparisons test (*p* < 0.05). Y axes represent mycelial area (mm^2^).

Of the tested VOCs, the five demonstrating the most significant inhibition were selected for further study at reduced doses, and all demonstrated significant inhibition when applied at reduced doses; 1‐octen‐3‐one: (*F*_7,16_ = 246.03; *p* < 0.001); 2‐octanone: (*F*_3,8_ = 42.29; *p* < 0.001); 3‐octanone: (*F*_4,10_ = 31.74; *p* < 0.001); 2‐pentylfuran: (*F*_5,12_ = 119.2; *p* < 0.001) and 2‐undecanone: (*F*_7,16_ = 91.65; *p* < 0.001) (Figure 4). 2‐octanone had a minimum inhibitory dose of 11.125 μM, 3‐octanone had a minimum inhibitory dose of 4.55 μM, 2‐pentylfuran had a minimum inhibitory dose of 4.55 μM and 2‐undecanone, 2.275 μM (Figure 5). The compound showing the greatest inhibition, 1‐octen‐3‐one, was still significantly inhibitory at a 100‐fold dilution (0.445 μM).

**FIGURE 6:**
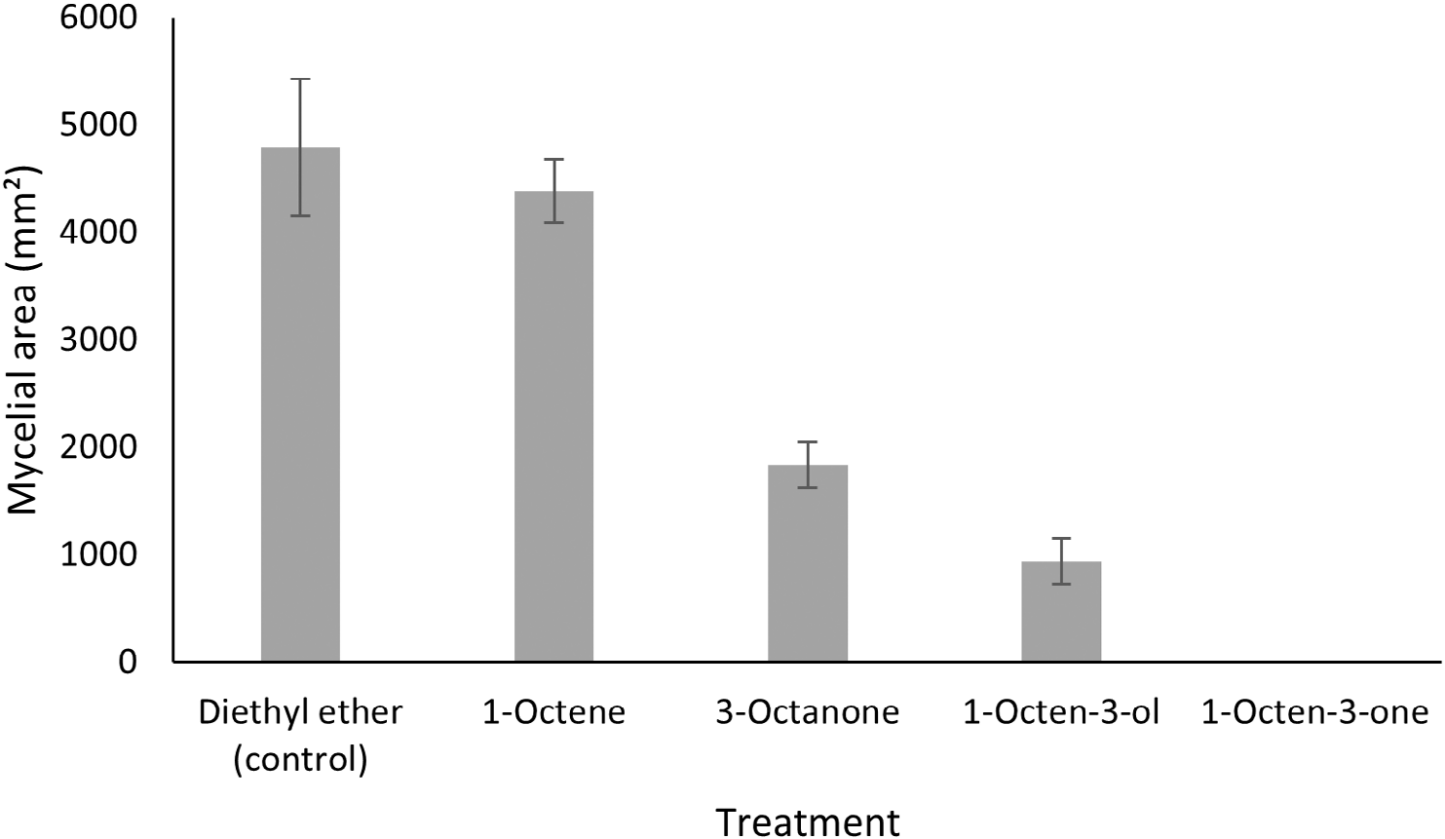
Antifungal activities of selected VOCs on the growth of *Sclerotinia sclerotiorum*, representing individual structural components of 1‐octen‐3‐one, at 45.5 μM. Bars represent the mycelial area of *S. sclerotiorum* upon exposure to each VOC (± SD) (*n* = 3). Different letters indicate significant differences between treatments according to Tukey's multiple comparisons test (*p* < 0.05). Y axis represents mycelial area (mm^2^).

**FIGURE 7:**
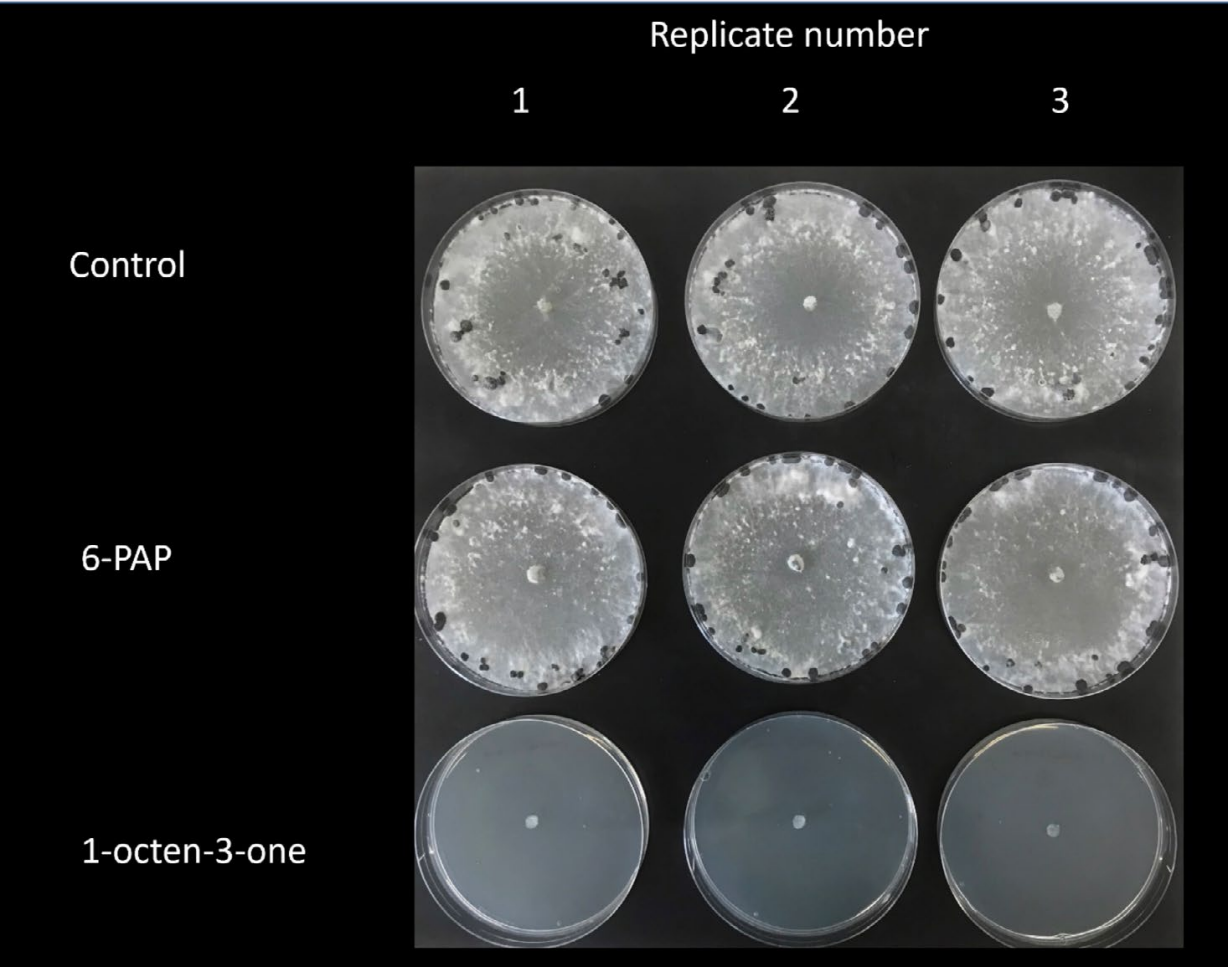
Antifungal activities of 6‐PAP and 1‐octen‐3‐one, on the growth of *Sclerotinia sclerotiorum,* at 45.5 μM.

To establish potential structural moieties required for antifungal activity of 1‐octen‐3‐one against *S. sclerotiorum*, compounds with similar structural features to both 1‐octene, 3‐octanone and (*RS*)‐1‐octen‐3‐ol were tested (Figure S4), revealing significant differences in inhibitory activities (*F*_4,10_ = 114.44; *p* < 0.001) (Figure 6). 1‐Octene demonstrated no significant inhibition of *S. sclerotiorum* relative to solvent controls (*p* > 0.05), whereas both 3‐octanone and (*RS*)‐1‐octen‐3‐ol showed significant inhibition of *S. sclerotiorum* relative to controls (*p* < 0.05). However, only 1‐octen‐3‐one demonstrated 100% inhibition. When *S. sclerotiorum* was removed from the shared atmosphere with 1‐octen‐3‐one, fungal growth of the pathogen was not restored 4 weeks after removing the pathogen from the headspace (Figure 7). This is consistent with 1‐octen‐3‐one having fungicidal activity at the tested dose.

## 4 Discussion

In this study, we showed induction of VOC production by *T. hamatum* GD12 occurs during co‐culture with the fungal pathogen *S. sclerotiorum*. This included VOCs not produced by *T. hamatum* when grown axenically. Whilst several VOCs were also produced in self‐challenged GD12 controls, the stimulation of VOC production in co‐cultures indicates an upregulation in production in the presence of the pathogen, some of which we demonstrate possess an antifungal role against *S. sclerotiorum*.

Many studies investigating VOC production from *Trichoderma* species utilise axenic fungal growth, and VOCs are predominantly assigned to alcohols, ketones, alkanes, furans, mono‐ and sesquiterpenes (Jeleń et al. 2014). Several low molecular weight compounds reported here as being produced by *T. hamatum* GD12 have been previously identified from other *Trichoderma* species, including 3‐octanone (Nemčovič et al. 2008; Stoppacher et al. 2010; Jeleń et al. 2014; Estrada‐Rivera et al. 2019; Speckbacher et al. 2020; da Silva et al. 2021) and 2‐octanone (Jeleń et al. 2014; Estrada‐Rivera et al. 2019; Speckbacher et al. 2020). To our knowledge, this is the first report of 1‐octen‐3‐one being produced by *T. hamatum*, although it was identified from *T. virens* (Li et al. 2018), and a range of other fungi (Pennerman et al. 2022). 6‐PAP is a characteristic *Trichoderma* VOC (Mendoza‐Mendoza et al. 2024), which produces a coconut aroma (Reithner et al. 2007; Stoppacher et al. 2010; Jeleń et al. 2014; Garnica‐Vergara et al. 2016; Estrada‐Rivera et al. 2019; Baazeem et al. 2021; da Silva et al. 2021). When comparing the *T. hamatum* GD12 VOCs identified via co‐culture with other studies, only 6‐PAP has been previously reported (Jeleń et al. 2014; Baazeem et al. 2021). However, directly comparing VOC diversity across other studies should be undertaken with caution as such studies employ different sampling techniques, which can introduce biases for certain compounds. For example, while many studies deploy SPME for headspace sampling, the diversity of fungal volatiles recovered depends on the type of fibre used (Stoppacher et al. 2010; Jeleń et al. 2014). Growth conditions of cultures will also likely vary across studies, which can influence VOC production from *Trichoderma*, including age of cultures (Lee et al. 2015), relative humidity and temperature of growth conditions (Polizzi et al. 2011), as well as media composition (Zhang et al. 2014; González‐Pérez et al. 2018). Intraspecific differences in VOC production have been observed for *T. hamatum*, as well as within other *Trichoderma* species. This likely relates to fungal evolution/adaptation to different geographical regions or ecological niches from which they were isolated (Jeleń et al. 2014; Lee et al. 2016), but also highlights the power of geographical/niche adaptation to drive the evolution of novel antifungals. Taken together, a range of factors can account for variation in VOC production by *Trichoderma*, both inter‐ and intra‐specifically.

Co‐cultivation with *S. sclerotiorum* revealed significant quantitative and qualitative changes in VOC production compared to the VOCs produced by *T. hamatum* GD12 in self‐challenged controls. This induction was greatest 17 dpi, consistent with other studies (Hynes et al. 2007). Two compounds which were identified using synthetic standards, 2‐pentylfuran and 2‐octanone, were biosynthesised *de novo* during the interaction between GD12 and *S. sclerotiorum*. Whilst it is difficult to ascribe the production of this compound to either GD12 or *S. sclerotiorum*, 2‐pentylfuran has previously been reported as being produced by *T. hamatum* from axenic cultures, with *T. atroviride* and *T. viridescens* producing 2‐octanone (Jeleń et al. 2014). Many of the upregulated compounds in our study are of the sesquiterpene‐like class, which have previously been observed during physical fungal‐fungal interactions (Hynes et al. 2007; Guo et al. 2019; Rajani et al. 2021), including *T. hamatum* when challenged with the ectomycorrhizal fungus *Laccaria bicolor* (Guo et al. 2019) and *Trichoderma* in confrontation with *Sclerotium rolfsii* and *Macrophomina phaseolina* (Sridharan et al. 2020). Sesquiterpenes are a well‐known class of compounds involved in chemical signalling, and have been isolated across a range of *Trichoderma* species, including *T. hamatum* (Ma et al. 2021), *T. brevicompactum* (Shi et al. 2020), *T. virens* (Shi et al. 2018, 2021; Hu et al. 2019), *T. longibrachiatum* (Du et al. 2020; Wang et al. 2022), *T. asperellum* (Ding et al. 2012), and *T. citrinoviride* (Liu et al. 2020). Many of these sesquiterpene‐like compounds possess antifungal activities against a range of phytopathogenic fungi, bacteria and marine phytoplankton. As well as their antimicrobial roles, microbial sesquiterpenes have a range of other biological activities including signalling, host growth promotion and defence (Avalos et al. 2022). The upregulation of unknown sesquiterpenes during co‐culture of *T. hamatum* and *S. sclerotiorum* could indicate a biological role for these compounds, and future work aims to isolate and identify these compounds to determine their role in the antagonistic response against *S. sclerotiorum*, and potential for integrating into biocontrol strategies.

6‐PAP dominated the VOC profile of *T. hamatum* GD12 co‐cultured with *S. sclerotiorum*, relative to self‐challenged *T. hamatum* GD12 cultures, corroborating previous work which found significant increases in 6‐PAP production when *T. harzianum* was co‐inoculated with *R. solani* (Serrano‐Carreón et al. 2004; Flores et al. 2019). Whilst significant increases in 6‐PAP production by *T. hamatum* GD12 in co‐culture were observed, no antifungal activity was observed when 6‐PAP was applied in the inverted plate assay setup (Figure S2). However, several studies demonstrate an inhibitory role for 6‐PAP against a range of fungal pathogens, including *Fusarium* species (Scarselletti and Faull 1994; El‐Hasan et al. 2008; Jeleń et al. 2014; Rao et al. 2022), *Botrytis cinerea* (Pezet et al. 1999), *Cylindrocarpon destructans* (Jin et al. 2020), and *Rhizoctonia solani* (Scarselletti and Faull 1994), when the compound was in contact with the pathogens. Recent work has identified a type I polyketide synthase (Pks1) which is responsible for 6‐PAP biosynthesis in *T. atroviride*, and when deleted, a loss of antagonism was observed against *B. cinerea* and *R. solani* in confrontation assays (Flatschacher et al. 2025). These studies indicate that 6‐PAP may require direct contact for effective antifungal activity. As well as 6‐PAP upregulation, 2‐octanone production was significantly upregulated in co‐culture treatments, indicative of *T. hamatum*–*S. sclerotiorum* antagonism. 2‐octanone upregulation has also been observed during the interaction between the fungal pathogen *Setophoma terrestris* and the beneficial soil bacteria *Bacillus subtilis* (Albarracín Orio et al. 2020), as well as the interaction between *T. atroviride* and *F. oxysporum* (Speckbacher et al. 2021), indicating a broader spectrum role for this compound in antagonistic fungal interactions. With several bioactive VOCs now identified, a crucial next step is to assess whether these compounds exhibit autotoxicity toward *T. hamatum* GD12. In parallel, transcriptomic studies of *T. hamatum* GD12 during its interaction with *S. sclerotiorum* will provide insight into the gene clusters responsible for antifungal VOC biosynthesis.

Many of the VOCs produced by *T. hamatum* GD12 during self‐challenge and in co‐cultures with *S. sclerotiorum* show significant antifungal activity against *S. sclerotiorum*. The antifungal role of 2‐octanone demonstrated here is in agreement with the inhibition of the soil fungal pathogen *Setaphoma terrestris*, which could indicate broad‐spectrum inhibitory activity against a range of fungal pathogens (Albarracín Orio et al. 2020). Similarly, 2‐heptanone has previously shown significant inhibition against *Curvularia lunata* (Xie et al. 2020) and *Alternaria solani* (Zhang et al. 2020). Here, we report only moderate antifungal activity of 2‐heptanone at the highest tested dose relative to other compounds, which may relate to differences in doses tested across the studies, or specificity in the antifungal activity of 2‐heptanone against different pathogenic species. Alternatively, it may reflect that the antifungal role could be derived from 2‐octanone via further modifications that may occur in a more complex soil microbiome, as opposed to our two‐component experimental system. Specificity of antifungal activity has been observed for 2‐undecanone, which has shown an inhibitory role against *Verticillium dahlia*, *F. oxysporum*, *B. cinerea* , and *Monilinia* spp., but not *Penicillium* spp. (Calvo et al. 2020) or *Rhizopus stolonifer* (Carter‐House et al. 2020). Having identified a range of antifungal compounds, determining the modes of action of the antifungal activities against *S. sclerotiorum* is an important next step. Furthermore, whilst the inhibitory properties of several *T. hamatum* VOCs have been demonstrated against *S. sclerotiorum*, it is important to establish that compounds at their inhibitory doses do not have phytotoxic effects. Interestingly, 2‐pentylfuran, which was upregulated in the presence of *S. sclerotiorum* and showed an antifungal role against the pathogen, has also demonstrable plant growth‐promoting capabilities (Zou et al. 2010). Thus, 2‐pentylfuran could potentially be a promising candidate to replace synthetic chemical inputs due to its ability to inhibit fungal pathogens without compromising plant growth.

To our knowledge, this is the first report demonstrating an antifungal role for 1‐octen‐3‐one against a pathogen. Although 1‐octen‐3‐one was not significantly upregulated in co‐cultures compared to monocultures, its antifungal activity remains noteworthy and may suggest constitutive production. When structurally related compounds (1‐octene, 3‐octanone, (*R,S*)‐1‐octen‐3‐ol) were tested for their antifungal activity at equivalent doses, 3‐octanone and (*RS*)‐1‐octen‐3‐ol demonstrated significant inhibition of *S. sclerotiorum*, whereas absolutely no growth of *S. sclerotiorum* occurred when exposed to 1‐octen‐3‐one. It is thus possible that fungicidal activity may be enhanced via Michael‐type acceptance by the α, β‐unsaturated carbonyl structure within the latter compound. Several of the strobilurin class of fungicides (fungicides derived from *Strobilurus* spp., Nofiani et al. 2018) also contain a conjugated ketone and alkene moiety. Findings here are contrary to those reported by Xiong et al. (2017), who found significant inhibition of *F. tricinctum* and *F. oxysporum* treated with 1‐octen‐3‐ol, but no inhibition when fungi were treated with 1‐octen‐3‐one (Xiong et al. 2017). However, VOCs were administered differently in each experiment, making cross‐comparison difficult. VOCs tested by Xiong et al. were supplemented into growth medium and in direct contact with *Fusarium* species, whereas tested VOCs here were physically separated from *S. sclerotiorum*, suggesting that the antifungal effect of 1‐octen‐3‐one works at a distance. Comparative studies directly comparing the methods used in this study compared to Xiong et al. would be required to confirm this. An important consideration is that, in both studies, (*RS*)‐1‐octen‐3‐ol was tested as a racemic mixture, although previous work has shown chirality can impact its antifungal activity (Yin et al. 2019). Whilst 1‐octen‐3‐one shows an inhibitory role against *S. sclerotiorum*, future work should determine the role of the compound on plant growth. For example, 1‐octen‐3‐one exposure significantly inhibits *Arabidopsis* growth and development (Lee et al. 2019), therefore future work should focus on determining which dose of 1‐octen‐3‐one can inhibit *S. sclerotiorum* without compromising plant growth. As compounds identified here demonstrate in vitro antagonism against *S. sclerotiorum*, immediate priorities will be to test these compounds against *S. sclerotiorum*, individually and in combinations, using peat microcosms under glasshouse conditions. These data will inform future open field trials, t examine their biological activities under more agriculturally relevant conditions.

In conclusion, this study suggests a role for volatile chemical signalling during the antagonistic response of *T. hamatum* GD12 against *S. sclerotiorum* and shows that certain *Trichoderma*‐derived VOCs play an inhibitory role against the pathogen. Specifically, we identify 1‐octen‐3‐one as a potential novel antifungal VOC. Further glasshouse and field tests with antifungal compounds identified here are required to determine whether they inhibit pathogens at a larger scale under more agriculturally relevant conditions. Whilst several of these compounds have been identified here, or previously described, many *T. hamatum* compounds upregulated on confrontation with fungal plant pathogens remain to be identified and characterised.

## Author Contributions

Conceptualisation: M.A.B., M.R.G., C.R.T. Data curation: G.A.T., J.V., J.C.C., M.A.B. Formal analysis: G.A.T., J.C.C., J.V., M.A.B., D.M.W. Funding acquisition: M.A.B., C.R.T., M.R.G. Investigation: M.A.B., G.A.T., M.R.G., C.R.T., J.V., D.M.W. Methodology: M.A.B., G.A.T., M.R.G., C.R.T., J.S. Project administration: M.A.B., M.R.G., C.R.T. Original draft: G.A.T., M.A.B., D.M.W. Reviewing and editing: G.A.T., M.A.B., D.M.W., J.V., C.R.T., M.R.G., J.S., J.C.C.

## Conflicts of Interest

The authors declare no conflicts of interest.

## Supporting information


**Data S1:** Supporting Information.

## Data Availability

The data that support the findings of this study are openly available at https://doi.org/10.23637/0dugoxf0 (Thomas et al. 2025).
