# Supplementary material for: Inducible Volatile Chemical Signalling Drives Antifungal Activity of Trichoderma hamatum GD12 During Confrontation With the Pathogen Sclerotinia sclerotiorum
Source: Environ Microbiol Rep. 2025 Sep 25;17(5):e70192. doi: 10.1111/1758-2229.70192 (PMC12462542; doi:10.1111/1758-2229.70192)
Supplement: Supplementary file 1 — Data S1: Supporting Information. [file EMI4-17-e70192-s001.docx]

**Inducible volatile chemical signalling drives antifungal activity of *Trichoderma hamatum* GD12 during confrontation with the pathogen *Sclerotinia sclerotiorum***

Gareth A. Thomas^1,2*^, József Vuts^1^, David M. Withall^1^, John C. Caulfield^1^, John Sidda^3^, Murray R. Grant^3^, Christopher R. Thornton^2^, Michael A. Birkett^1^

1. Protecting Crops and the Environment, Rothamsted Research, Harpenden, AL5 2JQ
2. Biosciences, College of Life and Environmental Sciences, University of Exeter, Exeter, EX4 4QD
3. School of Life Sciences, University of Warwick, Coventry, CV4 7AL

*Corresponding author

Supplementary information

*Sclerotinia sclerotiorum* plug

45.5 µM of synthetic standard

Figure S1 | Inverted plate bioassay set up

Figure S2 | Assessment of the antifungal activity of 6-Pentyl-2H-pyran-2-one (6-PAP) on the growth of *S. sclerotiorum*. *S. sclerotiorum* was incubated with 6-PAP at 45.5 µM doses, and the inhibition rates were calculated relative to control plates (exposed to diethyl ether alone) after 3 days. Bars represent the mean mycelial area of *S. sclerotiorum* upon exposure to each VOC (± SD) (n=3).


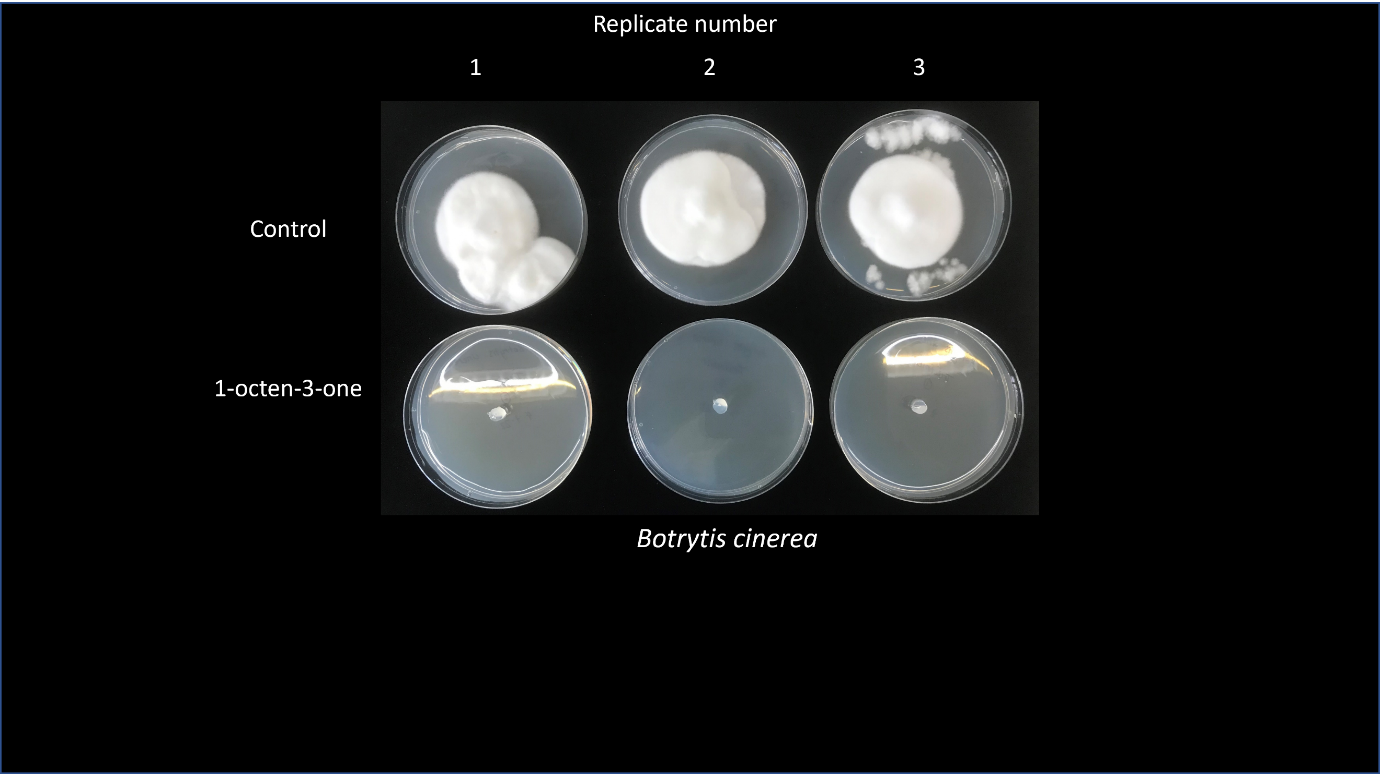


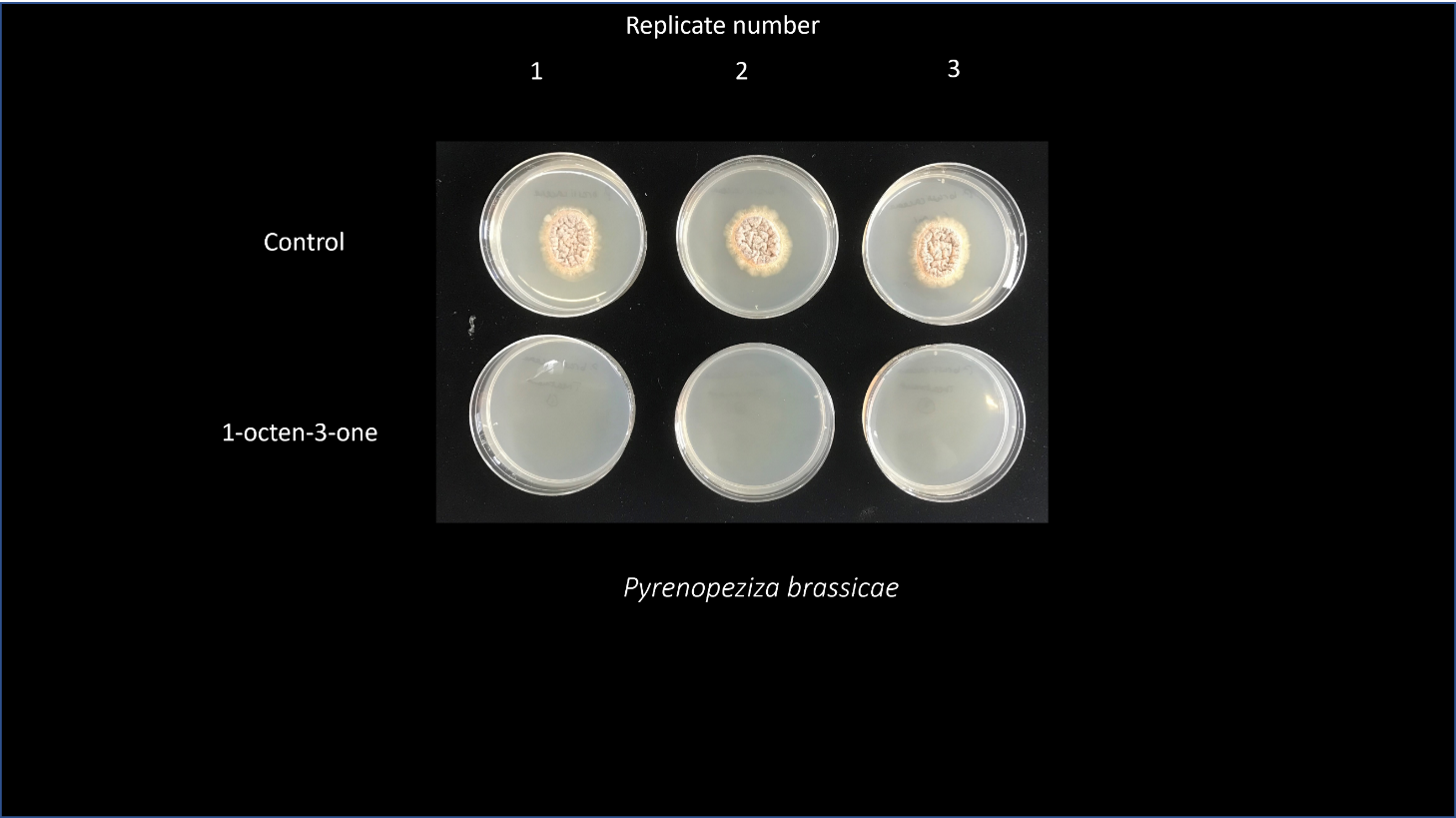


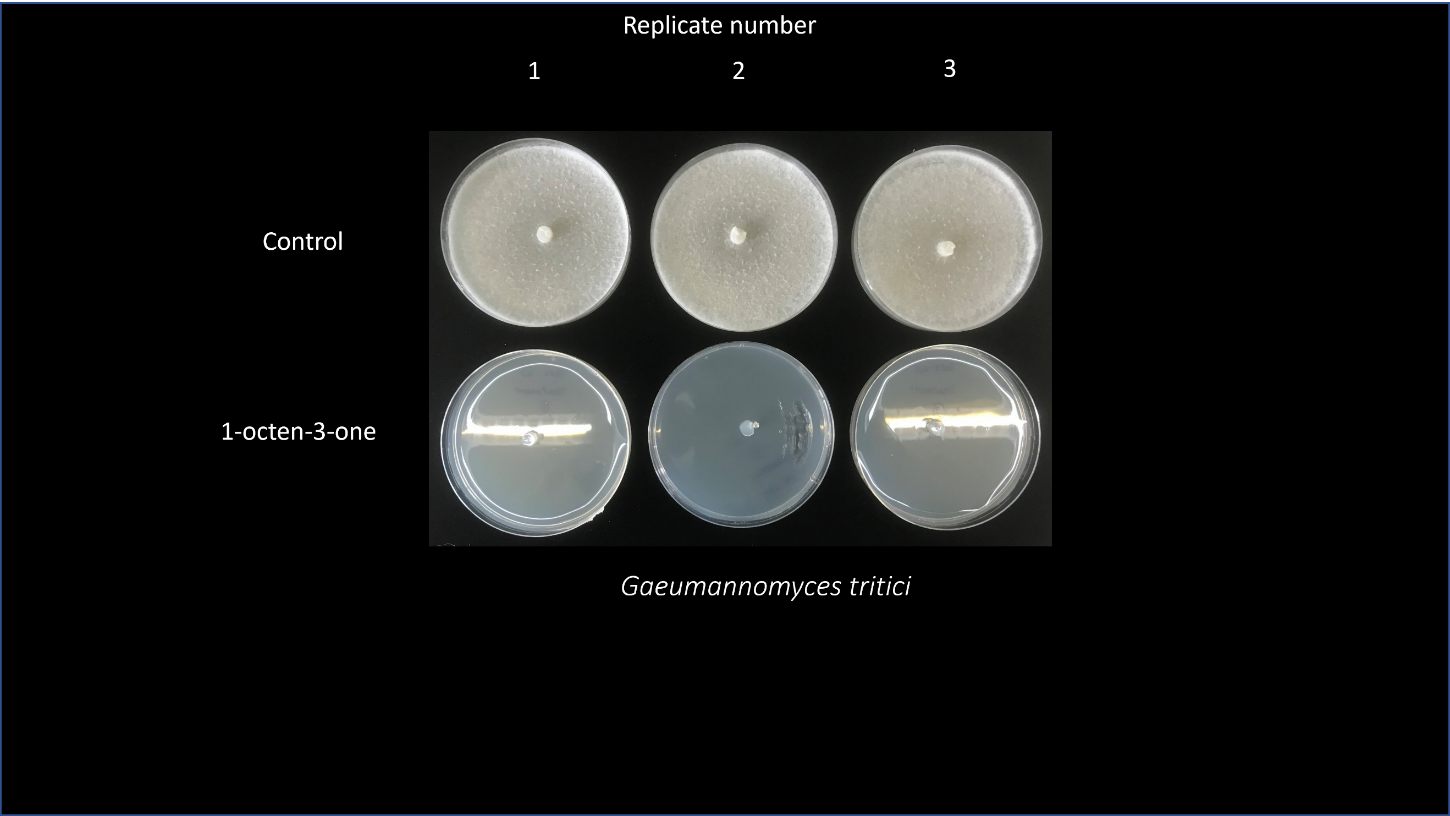


Figure S3 | Antifungal activities of 1-octen-3-one on the growth of *Botrytis cinerea*, *Pyrenopeziza brassicae* and *Gaeumannomyces tritici*. Fungal pathogens were incubated with 1-octen-3-one at a 45.5 µM dose.

Figure S4 | Compounds which make up the individual structural components of 1-octen-3-one.
